# Astaxanthin-Loaded Stealth Lipid Nanoparticles (AST-SSLN) as Potential Carriers for the Treatment of Alzheimer’s Disease: Formulation Development and Optimization

**DOI:** 10.3390/nano11020391

**Published:** 2021-02-03

**Authors:** Debora Santonocito, Giuseppina Raciti, Agata Campisi, Giovanni Sposito, Annamaria Panico, Edy Angela Siciliano, Maria Grazia Sarpietro, Elisabetta Damiani, Carmelo Puglia

**Affiliations:** 1 Department of Drug Science and Health, University of Catania, Viale Andrea Doria 6, 95125 Catania, Italy; debora.santonocito@outlook.it (D.S.); racitigi@unict.it (G.R.); agcampisi@gmail.com (A.C.); giovanni.sposito@hotmail.it (G.S.); panico@unict.it (A.P.); edysiciliano@hotmail.it (E.A.S.); mg.sarpietro@unict.it (M.G.S.); 2 Department of Life and Environmental Sciences, Polytechnic University of Marche, 60121 Ancona, Italy; e.damiani@univpm.it

**Keywords:** astaxanthin, stealth system, SLN, Alzheimer’s disease, ORAC, parenteral administration

## Abstract

Alzheimer’s disease (AD) is a neurodegenerative disorder associated with marked oxidative stress at the level of the brain. Recent studies indicate that increasing the antioxidant capacity could represent a very promising therapeutic strategy for AD treatment. Astaxanthin (AST), a powerful natural antioxidant, could be a good candidate for AD treatment, although its use in clinical practice is compromised by its high instability. In order to overcome this limit, our attention focused on the development of innovative AST-loaded stealth lipid nanoparticles (AST-SSLNs) able to improve AST bioavailability in the brain. AST-SSLNs prepared by solvent-diffusion technique showed technological parameters suitable for parenteral administration (<200 nm). Formulated nanosystems were characterized by calorimetric studies, while their toxicological profile was evaluated by the MTT assay on the stem cell line OECs (Olfactory Ensheathing Cells). Furthemore, the protective effect of the nanocarriers was assessed by a long-term stability study and a UV stability assay confirming that the lipid shell of the nanocarriers was able to preserve AST concentration in the formulation. SSLNs were also capable of preserving AST’s antioxidant capacity as demonstrated in the oxygen radical absorbance capacity (ORAC) assay. In conclusion, these preliminary studies outline that SSLNs could be regarded as promising carriers for systemic administration of compounds such as AST aimed at AD treatment.

## 1. Introduction

Alzheimer’s disease (AD) is a neurodegenerative disorder characterized by cognitive impairments. The classical symptoms of the disease include gradual deterioration of memory and language. This disease is characterized by the presence of extracellular amyloid-β (Aβ) plaques and intraneuronal tau neurofibrillary tangles in the cerebral cortex [1]. The main cause of the pathogenetic process of AD consists in a marked oxidative stress at the level of the encephalic cells affected by the neurodegenerative process [2]. Nowadays, there is no effective pharmacotherapy for the treatment of AD. Recent research and epidemiological studies indicate that potentiating the central nervous system’s (CNS) antioxidant capacity, through the administration of antioxidant compounds, such as astaxanthin, could represent a promising strategy for the treatment of neurodegenerative disorders such as AD [3,4].

**Figure 1:**
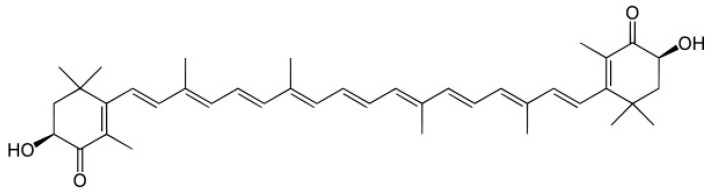
Chemical structure of astaxanthin (AST).

Astaxanthin (3,3′-dihydroxy-β, β′-carotene-4,4′-dione; AST) is a fat-soluble orange-red colored pigment extracted from *Haematococcus pluvialis*, a unicellular biflagellate green microalga. It is a carotenoid belonging to the family of the *Xanthophylls* and it is responsible for the coloring of some fish, including salmon and shellfish [5]. AST exhibits powerful antioxidant activity and possesses many valuable therapeutical functions, such as anti-aging, anticancer [6,7,8], and neuroprotective effects [9]. The remarkable scavenging activity of AST is due to its chemical structure which traps radicals in the polyene chain (Figure 1). It is a highly lipophilic molecule, but the presence of two terminal hydrophilic groups determines a slight solubility in water.

This dual nature and its small size allow it to easily permeate cell membranes. In fact, it is a carotenoid that carries out its antioxidant and anti-inflammatory actions at the level of the eyes, the brain and the CNS, as it manages to cross the blood–brain barrier and the blood–retinal barrier. Many studies have indicated that AST is a promising biologically active compound for the treatment of AD. Wang and coworkers reported that AST protected neuroblastoma cells against Aβ-induced oxidative cell death through induction of the antioxidant enzyme HO-1 expression [10]. In particular, AST is able to prevent amyloid-β (Aβ) accumulations through the formation of hydrogen bonds and Van der Waals interactions with Aβ [11,12,13,14,15]. Furthermore, it is reported that AST is able to reduce the ischemia-related injury in brain tissue, mainly through the inhibition of oxidative stress [16].

Although AST possesses a high pharmacological potential, it has no applications in the clinical setting due to poor drug stability. The presence of C=C double bonds in the chemical structure of carotenoids makes the molecule sensitive to oxidation, to air and to photo-oxidation. All these factors can cause their conversion into a mixture of stereoisomers (with trans-cis isomerization). Furthermore, AST is sensitive to thermal degradation and it is poorly water soluble [17]. Therefore, innovative strategies are required in order to overcome the limits of this interesting natural active compound, thus allowing its exploitation in the therapeutic field [18,19].

Recently, nanomedicine, in particular the use of solid lipid nanoparticles (SLNs), is attracting considerable attention due to its potential advantages for enhancing pharmacokinetics as well as pharmacodynamics activity of plant-derived constituents. SLNs are colloidal carriers made from solid biodegradable lipids (GRAS) stabilized by surfactants, with a mean diameter ranging from 50 to 1000 nm. SLN present several advantages over other colloidal carriers (liposomes and polymeric nanoparticles) such as higher drug stability, incorporation of both hydrophilic and lipophilic drugs, absence of toxicity, easy large-scale production, and the possibility of lyophilization. The composition of these nanoparticles, along with their characteristics, make them ideal for the carrying/delivery of sensitive bioactive compounds, protecting them against chemical degradation and also facilitating their application in various administration routes [20,21,22,23]. The encapsulation of drugs into these nano-carriers increases their solubility, stability, cell uptake, specificity, tolerability, and therapeutic index. Therefore, these carriers are suitable for almost all routes of drug administration, including the parenteral route, one of the most commonly used routes for drug administration at the CNS level. Unfortunately, the use of SLNs through systemic administration is limited by the opsonization process, one of the most important mechanisms used by the immune system to fight pathogens [24], which is responsible for their short half-life (3–5 min) after intravenous administration [25,26,27].

One of the most used strategies to prolong the systemic residence of these carriers is based on the modification of the nanoparticle surface to achieve ‘stealth’ systems able to overcome the defense line represented by macrophages, through a biomimetic mechanism [28]. One of strategies used to realize ‘stealth’ systems is to coat the nanoparticle surface with surfactants, such poloxamers [29]. This hydrophilic coating modifies the SLN biodistribution, improving blood circulation times and deposition in non-RES (reticuloendothelial system) organs [30]. Among the different surfactants used to modify particle distribution, polysorbate 80 (p80) is one of the most effective surfactants for enhancing concentration in the CNS [31].

The aim of the present work was to formulate stealth lipid nanoparticles loaded with AST (AST-SSLNs) as a potential strategy to increase AST concentration in CNS. The obtained formulation was characterized in terms of technological parameters (PDI = polydispersity index, mean particle size and ZP = zeta potential), cytotoxicity of raw materials and formulated nanosystems, and stability over time. The screening of lipid matrix was carried out on human dental pulp stem cells (hDPSCs) transdifferentiated into neuronal stem cells (NSCs), while the cytotoxicity of AST-SSLN was evaluated on primary olfactory ensheathing cells (OECs) and on MCF-7 cell line, used as cancer cell type [32,33]. Furthermore, the antioxidant effect of AST, once loaded in SSLN, was evaluated through the ORAC assay.

## 2. Materials and Methods

### 2.1. Materials

Compritol 888 ATO (MW 414.7 g/mol), a mixture of mono-, di-, and triglycerides of behenic acid, and Precirol ATO 5 (Glyceryl palmitostearate) were obtained from Gattefossè (Milan, Italy); Lecinol S-10, hydrogenated lecithin, was obtained from Nikko Chemical (Milan, Italy) and Lutrol F68 (MW 8400 g/mol) was provided by BASF ChemTrade GmbH (Burgbernheim, Germany). Astaxanthin (AST), Trolox, AAPH (2,2′-azobis(2-methylpropionamidine) dihydrochloride), stearic acid, and all reagents were purchased from Sigma-Aldrich (St. Louis, MO, USA). Fluorescein disodium salt was obtained from Acros Organics (Milan, Italy). Cytosine arabinoside, 1-(4,5-Dimethylthiazol-2-yl)-3,5-diphenylformazan (MTT), DMSO, and other chemicals were from Sigma-Aldrich (Milan, Italy). Polysorbate 80 was obtained from Polichimica S.r.l (Bologna, Italy) while collagenase type I, sodium pyruvate, trypsin/EDTA solution were from GIBCO, Thermo Fisher (Milan, Italy). Nerve growth factor (NGF) were purchased from GIBCO (Thermo Fisher, Milan, Italy). Basic fibroblastic growth factor (bFGF), human epidermal growth factor (hEGF) retinoic acid, dibutyryl cAMP (dbcAMP), and isobutyl methyl xanthine (IBMX) were purchased from MERK (Darmstadt, Germany). BDNF was purchased from PrepoTech (London, UK).

### 2.2. SLN Preparation

SLNs were prepared by *solvent-diffusion* technique, using stearic acid as lipid phase and Lutrol F68^®^ (Poloxamer 188) as surfactant. The lipid matrix most suitable for the encapsulation of AST was stearic acid [34]. The choice to use this lipid was further confirmed by the in vitro assay subsequently described.

Briefly, stearic acid (0.005 g) and AST (AST 4.2 mg) were solubilized in ethanol (1.2 mL) and maintained in a fluid state at 70 °C. The aqueous phase was constituted by hydroxypropylmethyl cellulose (0.05 g), soy lecithin (0.05 g), Lutrol F68 (0.05 g), and distilled water (5.8 mL). The melted lipid phase was dispersed in the hot (70 °C) aqueous phase by using a high-speed stirrer (Ultra-Turrax T25, IKA-Werke GmbH &Co. Kg, Staufen, Germany) at 15,000 rpm for 8 min, maintaining the temperature at least 10 °C above the lipid melting point. The obtained pre-emulsion was ultrasonified by using an ultrasonic processor (UP 400 S, Dr. Hielscher GmbH, Stuttgart, Germany) for 10 min. Then the hot dispersion was cooled in an ice bath for 5 min. In order to modify the surface of the nanoparticles, p80 (20% *w*/*w* with respect to the lipid weight) was added to the SLN stirring at 250 rpm for 30 min. Finally, the organic solvent was removed under vacuum. Blank SSLN were prepared by the same procedure without the addition of AST.

### 2.3. SSLN Characterization

SSLN average size (Z-Ave) and polydispersity index (PDI) were determined by photon correlation spectroscopy (PCS) using a Zetasizer Nano-ZS90 (Malvern Instrument Ltd., Worcs, UK), equipped with a solid state laser having a nominal power of 4.5 mW with a maximum output of 5 mW at 670 nm. The measurement method is based on the principle of dynamic light diffusion. Analyses were performed using a 90° scattering angle at 20 ± 0.2 °C. The zeta potential (ZP) is an indicator of the stability of a dispersed system. This parameter is determined using the electrophoretic light scattering (ELS) technique, which measures the electrophoretic mobility of particles in dispersion or in solution. Samples were prepared diluting 100 μL of SSLN suspension with 900 μL of distilled water and the measurements were recorded at 25 °C. Each value was measured at least in triplicate.

### 2.4. Morphological Study

AST-SLN and AST-SSLN morphology was evaluated by means of transmission electron microscopy (TEM) (Philips EM 400T microscope, Eindhoven, The Netherlands). TEM samples were prepared by deposition of a drop of diluted (100-fold) SLN and SSLNs suspensions on the surface of a 200 mesh Formvar^®^-coated copper grid (TAAB Laboratories Equipment, Ltd., Aldermaston, UK) and letting the drop evaporate at room temperature overnight. Specimens were prepared by deposition of diluted (100-fold) SLN and SSLNs suspensions onto an aluminum specimen stub covered with a double-sided adhesive carbon disk. After water vaporization at room temperature, samples were sputter coated with chromium prior to imaging (Quorum Q150T ES East Grinstead, West Sussex, UK). Coating was done at 120 mA for 30 s.

### 2.5. Determination of AST Loading

The unentrapped AST was determined by filtration using a Pellicon XL™ tangential ultrafiltration system (Millipore, Milan, Italy). This is equipped with a polyethersulfone Biomax 1000 membrane with a 1,000,000 molecular weight cut off (MWCO). An amount of the lyophilized nanoparticles was solubilized in dichloromethane and the AST content was measured by UV spectrophotometry at 492 nm (Lambda 52, Perkin Elmer, Boston, MA, USA). To validate the UV assay, calibration curves were performed on six solutions in the AST concentration range 10–100 μg/mL (r^2^ > 0.99). Each point represents the average of three measurements and the error was calculated as standard deviation (±SD). AST incorporation efficiency was calculated from Equation (1)

Drug recovery (%) = (Mass of active in nanoparticles/Mass of active fed to the system) × 100 (1)

Possible lipid interferences during UV determination of AST were also studied between the standard curves of AST alone and in the presence of lipids. The differences observed were within the experimental error, thus inferring that no lipid interference occurred.

### 2.6. In Vitro Release Study

The amount of AST released from SLN and SSLN was calculated through the permeability test. This test was carried out using vertical Franz cells consisting of a receptor and a donor compartment. The receptor compartment (4.5 mL) was filled with phosphate buffer solution (PBS, pH 7.4) and placed in a bath at 37 °C, under magnetic stirring. The testing sample (200 µL) was placed in the donor cell maintaining a complete and intimate contact with the surface of a cellulose acetate membrane (0.2 µm pore size, 25 mm diameter, Sartorius; Göttingen, Germany). At predetermined intervals, samples (200 µL) at each time point (0, 2, 4, 6, 8, 22, and 24 h) were taken from the receptor chamber and replaced with the same volume of PBS. The amount of AST permeated was analyzed by UV spectrophotometry at 492 nm.

### 2.7. Differential Scanning Calorimetry (DSC)

DSC analysis was carried out using a Mettler Toledo STAR^e^ system (Schwerzenbach, Switzerland) equipped with a DSC-822 calorimetric cell. A MettlerTA-STAR^e^ software (16.00 version) was used to acquire and to analyze data. The DSC was calibrated using indium (≥99.95% purity). The reference pan was filled with 120 μL of distilled water. Each sample (120 μL) was loaded into a 160 μL aluminum crucible, hermetically sealed and submitted to DSC analysis, under an atmosphere of dry nitrogen. DSC analysis was carried out using a heating scan from 5 °C to 85° C (2° C/min) and a cooling scan from 85 °C to 5 °C (4° C/min) at least three times [35].

### 2.8. Long-Term Stability

Mean particle sizes, PDI and ZP values of SLN and SSLN samples were monitored over time at intervals (time zero, one week, two weeks, three weeks, one month, and every month onwards up to six months). During storage, samples were maintained at room temperature and protected from light exposure.

### 2.9. In Vitro Study on AST-SSLN

#### 2.9.1. Cell Cultures: Treatment and MTT Assay

Human dental pulp stem cells (hDPSCs) were isolated from teeth of anonymous donors/patients, according to the ethical Committee of Azienda Ospedaliero-Universitaria “Policlinico-Vittorio Emanuele” (Catania, Italy) [36]. Briefly, freshly extracted teeth were opened and the dental pulp tissue was taken, fragmented, digested with 3 mg/mL collagenase type I for 1 h at 37 °C and centrifuged at 200× *g* for 15 min. Then, cells were suspended in DMEM, containing 15% FBS, 2 mM L-glutamine, 100 μg/mL streptomycin, 100 U/mL penicillin G, and 1 mM sodium pyruvate, at a final concentration of 1 × 10^6^/mL. Cell cultures were incubated at 37 °C in humidified atmosphere containing 5% CO_2_. The culture medium was replaced every 2–3 days. When the cell cultures reached about 85–90% confluency, they were subcultured at 1:4 density ratio by trypsinization and incubated at 37 °C in humidified atmosphere containing 5% CO_2_. After 6 days in vitro (DIV), the hDPSCs were neural differentiated (hNSCs) as previously reported [37], suspended in DMEM containing 10% FBS and 10 μM retinoic acid and maintained at 37 °C in humidified atmosphere containing 5% CO_2._ The culture medium was replaced every 2–3 days.

Primary OECs were isolated from rat pups (P2) olfactory bulbs as previously described [38]. Animals were housed in a light and temperature-controlled room (23 ± 1 °C, 50 ± 5% RH) with tap water and standard chow provided ad libitum. Experimental animal procedures were carried out according to the Italian Guidelines for Animal Care (D.L. 116/92 and 26/2014), which are in compliance with the European Communities Council Directives (2010/63/EU) and were approved by the Ethical Committee at the University of Catania (Catania, Italy). Efforts were made to minimize the number of animals used and their suffering. Briefly, pups were decapitated and the bulbs were removed. Then, they were digested by collagenase and trypsin. Trypsinization was stopped by adding DMEM supplemented with 10% heat inactivated FBS, 2 mM glutamine, streptomycin (50 μg/mL) and penicillin (50 U/mL). Cells were resuspended and plated in flasks fed with fresh complete medium. The antimitotic agent, cytosine arabinoside (5–10 M), was added 24 h later. To further purify them, OEC cultures were further processed by transferring the cells from one flask to a new one [39,40]. In the last passage, OECs were plated on 25 cm^2^ flasks and cultured in DMEM/FBS supplemented with a bovine pituitary extract. Cells were then incubated at 37 °C in humidified air containing 5% CO_2_ with fresh complete medium which was refreshed twice a week.

MCF-7 human breast cancer cells were suspended in DMEM containing 10% (*v*/*v*) FBS, 2 mM L-glutamine, 50 mg/mL penicillin (50 U/mL), and plated in 75 cm^2^ flasks at a final density of 2 × 10^6^ cells. The cell cultures were then incubated at 37 °C in humidified atmosphere and the medium was replaced every 2 or 3 days. When the cultures were about 80–85% confluent, they were subcultured at 1:4 density ratio by trypsinization and incubated at 37 °C in humidified atmosphere containing CO_2_.

#### 2.9.2. Treatment of Cell Cultures

The purified OEC, hNSC and MCF-7 cell cultures were suspended in their specific complete culture medium and seeded into in 96-multiwell plates at a final density of 1 × 10^4^ cells and incubated at 37 °C in a humidified atmosphere and CO_2_ (95–5%). hNSCs were exposed for 24 h to 3.5 μM stearic acid, 4.5 μM Compritol, 16 μM Precirol. OECs were exposed for 24 h to blank SSLN (0.1, 0.2, and 1 μM), AST-SSLN (0.1, 0.2, and 1 μM). MCF-7 cell line cultures were exposed for 24 h to blank SSLN (0.2, 0.4, and 1 μM), AST-SSLN (0.2, 0.4, and 1 μM). The stock solutions of PBS and ETOH were prepared at 1% *v*/*v*. One µL of each was added to 200 µL of each well of a 96-multiwell plate, having a final concentration of 0.005% *v*/*v*. Cells were also treated with the corresponding volume of ethanol (ETOH 0.005%) used to solubilize AST, at a final ethanol concentration of 0.005%.

#### 2.9.3. MTT Assay

In untreated and treated OEC, hNSC, and MCF-7 cell cultures, 20 μL of 0.5% MTT solution were added to each multiwell as previously reported [27]. After 2 h of incubation at 37 °C, the supernatant was removed, replaced with 100 μL DMSO and incubated at 37 °C for 1 h. The optical density of each well sample was measured on a microplate spectrophotometer reader (Titertek Multiskan; Flow Laboratories, Helsinki, Finland) at 570 nm. Results are expressed as a percentage of the control (PBS and Ethanol), taken as 100%, to normalize the different values obtained.

### 2.10. Oxygen Radical Absorbance Capacity (ORAC) Assay

The antioxidant capacity of AST, free and encapsulated into SLN and SSLN, was measured using ORAC assay. During this test, the decay in fluorescein fluorescence due to peroxyl-radical formation by the peroxyl radical generator AAPH (2,2′-azobis 2- amidinopropane, dihydrochloride) is monitored over time. FL solution containing AAPH was used as the positive control and FL solution without AAPH as negative control. The assay was performed at 37 °C on a VICTOR Wallac 1420 Multilabel Counters fluorimeter (Perkin Elmer, Boston, MA, USA) set with excitation filter 540 nm and emission filter 570 nm. Fluorescein (FL) and AAPH were used at 12 nM and 100 mM, respectively. AST was solubilized in ethanol (12.5 µM), while SLN and SSLN formulations were diluted at the same concentration with phosphate buffer. After adding AAPH, the fluorescence was monitored for 30 h. Trolox was used as reference compound.

### 2.11. UV Stability Assay on AST-SSLN

In order to evaluate the photostability of lipid nanoparticles against UVA radiation, 600 μL of each sample were dispensed into a well of a 24-well plate, covered with a quartz plate and placed on a brass block embedded on ice for UVA exposure. Samples were exposed from above to 15 min UVA irradiation which corresponds to an incident dose of UVA of 275 kJ/m^2^, i.e., the dose approximately equivalent to about 90 min of sunshine [41]. As irradiating source, a commercial UVA sun lamp was used as previously described [42]. After exposure, 200 μL were taken and extracted in 1 mL ethyl acetate by vortexing for 2 min, followed by brief centrifugation to separate the organic layer from the water phase. The UV/Vis spectrum of the organic solution was then measured on a UV Varian Cary 50 spectrophotometer (Agilent Technologies Italia S.p.A., Milan, Italy) against a blank containing ethyl acetate.

### 2.12. Statistical Analysis

Data were statistically analyzed using one-way analysis of variance (ANOVA) followed by a post hoc Holm–Sidak test to estimate significant differences among groups. Data are reported as mean ± SD of at least three independent experiments performed in duplicate, and differences between groups were considered to be significant at * *p* < 0.05.

## 3. Results and Discussion

### 3.1. Screening of Lipid Matrix

The choice of the most suitable lipid matrix to formulate AST-SSLN was first determined using the MTT assay, since this test proved useful for revealing the potential cytotoxic effects of excipients on adult primary stem cells (primary adult stem cell human dental pulp, DPSCs) [36,43]. DSPCs isolated from human dental pulp and differentiated into neurons, derive from the embryonic neural crest and represent a useful source of primary cells for modeling neurological disorders at the molecular level [44,45]. This lipid screening was done by preparing blank SSLN using different lipid matrices such as stearic acid, Compritol 888 ATO and Precirol 5 ATO. Subsequently, the effect of these formulations on cell viability of the neuronal cell line DPSC was investigated, since these stem cells possess protective effects against models of neurodegenerative diseases including AD [43,46]. In preliminary experiments, the possible interactions between SSLN and MTT in the absence of cells were tested, in order to evaluate if they interact with the assay. The results obtained did not show interactions between SSLN and MTT.

**Figure 2:**
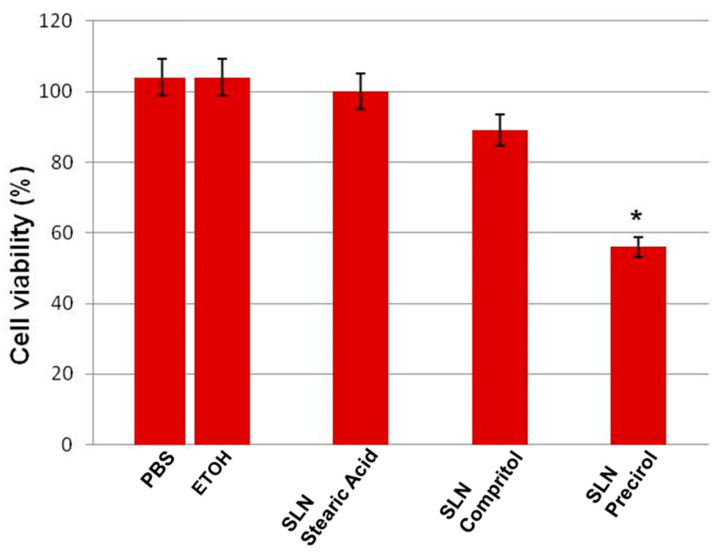
Percentage viability of primary human NSCs exposed to 0.005% of PBS (phosphate buffer saline solution), 0.005% ETOH (ethanol), stearic acid (3.5 µM), Compritol 888 ATO (4.5 µM), and Precirol 5 ATO (16 µM) for 24 h. Results are expressed as the mean ± S.D. of the values of five independent experiments performed in triplicate. * *p* < 0.05 vs. controls (PBS and ETOH). SLN: solid lipid nanoparticles; NSCs: neuronal stem cells.

As shown in Figure 2, no significant differences between PBS and ethanol-treated NCSs were observed, hence they were both used as controls. The percentage of inhibition of cell viability was compared with the controls taken as 100%. In NSCs exposed for 24 h to stearic acid and Compritol 888 ATO no significant changes in the percentage of cell viability were found. In contrast, the treatment of the cells with Precirol 5 ATO induced a significant reduction in the percentage of cell viability. Although Compritol 888 ATO and stearic acid showed a similar pattern, we chose to formulate AST-SSLN with stearic acid as lipid matrix for SSLN characterization due also to the high affinity of AST toward this lipid excipient [34].

### 3.2. Formulation and Characterization of AST-SSLN

AST-SSLN were formulated by the solvent-diffusion technique using stearic acid as lipid phase and Lutrol F68^®^ (Poloxamer 188) as surfactant. The method used has proven to be valid and reproducible despite the instability of AST. As reported in Table 1, DLS (dynamic light scattering) data showed that nanoparticles have a mean diameter around 110–150 nm and a good homogeneity, while the Z potential values indicate good stability of the nanodispersions. Furthermore, the mean particle size of SLN was lower (about 20 nm) compared to SSLN, likely due to the p80 layer which fits into the surface of the nanoparticle [29]. As regards to drug loading determination (DR%), the encapsulation efficiency of AST was above 90%.

**Figure 3:**
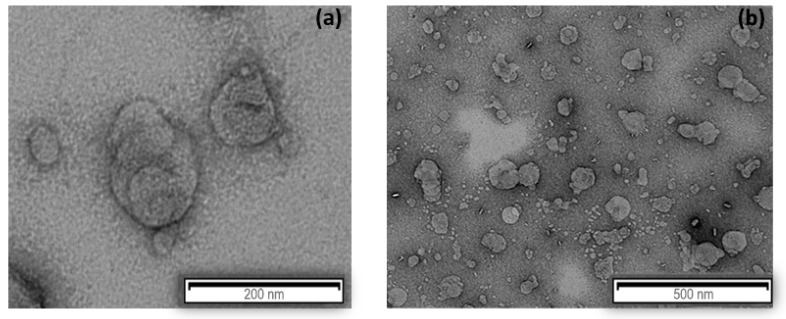
Transmission electron microscopy images of (**a**) AST-SLN and (**b**) AST-SSLN.

The morphology of AST-SLN and AST-SSLN were determined using TEM (transmission electron microscopy) (Figure 3). TEM images showed that the lipid nanoparticles had a spherical appearance with a particle size suitable for parenteral administration (<200 nm). This particle size is in agreement with the DLS data.

### 3.3. In Vitro Release of AST

**Figure 4:**
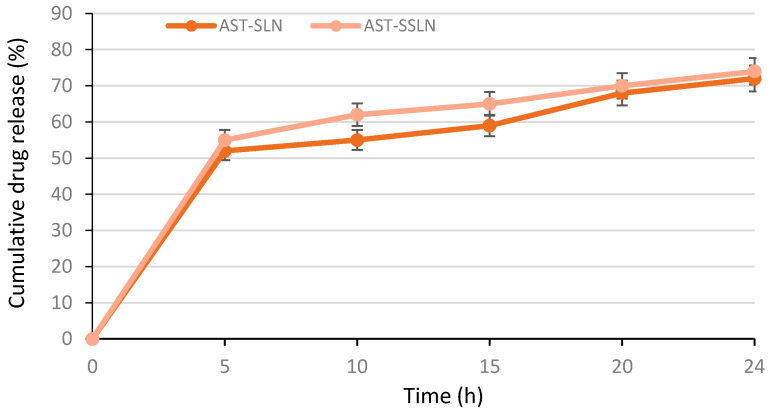
In vitro release profiles of AST from SLN and SSLN. Each point represents the mean value ± S.D. (*n* = 3).

The in vitro release profiles of AST from SLN and SSLN, as shown in Figure 4, follow a “two-step drug release” related to the distribution of the active compound in the SLN matrix [47]. In the first phase, AST present on the nanoparticle surface is released immediately, while in the second phase it is released slowly since it derives from the lipid core. Therefore, the encapsulation of AST into SLN could be an effective way to supply it continuously in the body. In this last phase, approximately 70% of encapsulated AST is released and both formulations show a similar behavior.

### 3.4. Differential Scanning Calorimetry (DSC)

**Figure 5:**
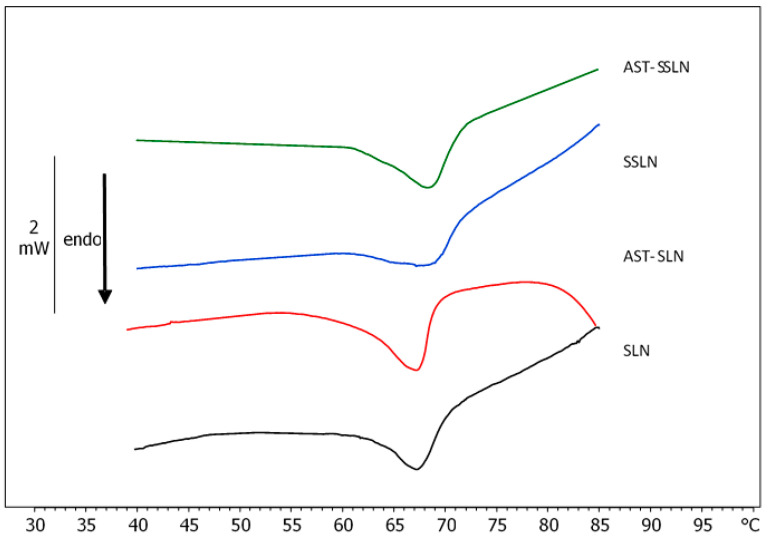
Calorimetric curves in heating mode, of SLN and SSLN unloaded and loaded with AST.

The thermodynamic characterization of a system is useful for obtaining information on the interactions among different components of the system. DSC detects the temperature-dependent heat capacity of a sample submitted to heating and/or cooling scans. This measure allows to obtain data on thermodynamic parameters, among which the transition temperature (T_m_) and enthalpy variation (∆H) are the most important [48,49,50], and by comparing the data from different samples, information about the components distribution and interactions can be obtained. The calorimetric curve of unloaded SLN is characterized by a main peak at about 67.5 °C (Figure 5). In the presence of AST, the calorimetric peak retains its shape and temperature, but it becomes larger; this can be due to a homogenous distribution of AST on the SLNs structure. AST could interact with the SLNs molecules stabilizing the system. The calorimetric curve of SSLN is characterized by a main peak at about 68.5 °C and a shoulder at lower temperatures. This is evidence of a non-homogeneous distribution of the SSLNs components and of phase separation occurring. For instance, p80 could preferentially localize in some parts of the SSLNs; in particular, there may be an internal zone devoid of p80 and an external one with p80. Hence, p80 could cover the SSLNs surface with some part of the molecule inserting inside it. In the SSLNs loaded with AST, the shoulder is still evident and the main peak becomes sharper with respect to SSLN alone. Since the sharpness of the peak is an index of the cooperativity among the molecules of the system, we can hypothesize that AST distributes in the SLNs with a stabilizing effect. The enthalpy variation was obtained by integration of the area under the transition peak. The results obtained show only slight differences among the samples with; the values ranging from 176.45 to 179.39 J/g. These results suggest that AST can act as “interstitial impurities” in the system, by intercalating among the lipids, causing T_m_ variations without ΔH differently to molecules that act as “substitutional impurities” taking the place of lipid molecules, and causing variations in T_m_ variation and ΔH [50].

### 3.5. Stability Studies on AST-SSLN

**Figure 6:**
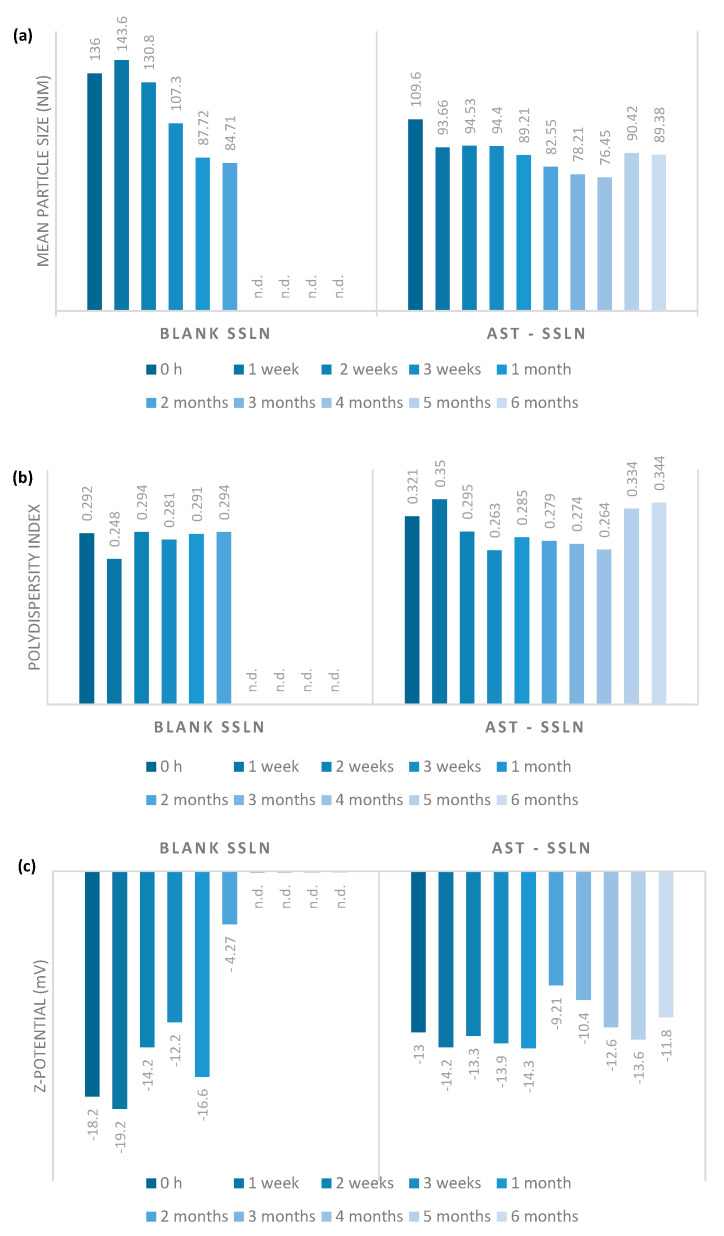
(**a**) Particle size, (**b**) polydispersity index (PDI), and (**c**) Z-potential of unloaded and AST-SSLN during storage at room temperature for 6 months. n.d. = not detectable.

Long-term stability of SLN and SSLN unloaded and loaded with AST were monitored for 6 months during storage at room temperature in order to obtain information on their long-term stability (Figure 6). The data shows that nanoparticles have an acceptable long-term stability after 6 months of storage. Furthermore, AST-SSLNs are more stable than unloaded SSLNs; this is most probably due to AST antioxidant capacity to preserve the system against instability phenomena [51]. The same results were obtained with SLNs (data not shown).

### 3.6. Cell Viability

In order to evaluate the possible toxic effects of blank and AST-SSLN, the MTT assay was carried out. The cytotoxicity studies were carried out using different cell lines, in particular a stem cell line, OECs and a cancer cell line, MCF-7 that differ in the characteristics of their cell membrane. We used OECs because they represent a particular glial cell type exhibiting phenotypic properties with both Schwann cells (SCs) and astrocytes [52]; they protect the small unmyelinated axons of olfactory receptor neurons driving them towards their correct position from the basal lamina of the epithelium to the olfactory bulb [53]. In addition, this cell type might represent an interesting model for the treatment of AD [32]. MCF-7 cell line represents a human breast cancer cell line commonly used as a model for cancer investigations.

**Figure 7:**
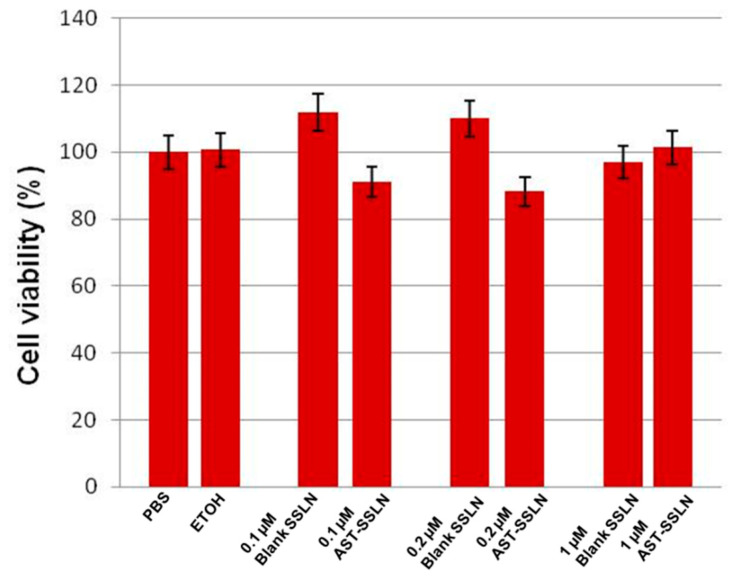
Percentage of cell viability in OECs exposed to 0.005% of PBS (phosphate buffer saline solution), 0.005% ETOH (ethanol), Blank-SSLN and AST-SSLN (0.1, 0.2, and 1 μM) for 24 h. All the values shown represent the mean ± S.D. of three separated experiments performed in triplicate.

Figure 7 shows the effect of the treatment of OECs for 24 h with blank-SSLN and AST-SSLN at different concentrations (0.1, 0.2, and 1 μM). No significant differences between PBS and ethanol-treated OECs were found, hence they were both used as controls. The percentage of inhibition of cell viability was compared with the controls taken as 100%. In the normal cells (OECs) no significant changes were found between the different concentrations tested and their respective blank-SSLNs.

**Figure 8:**
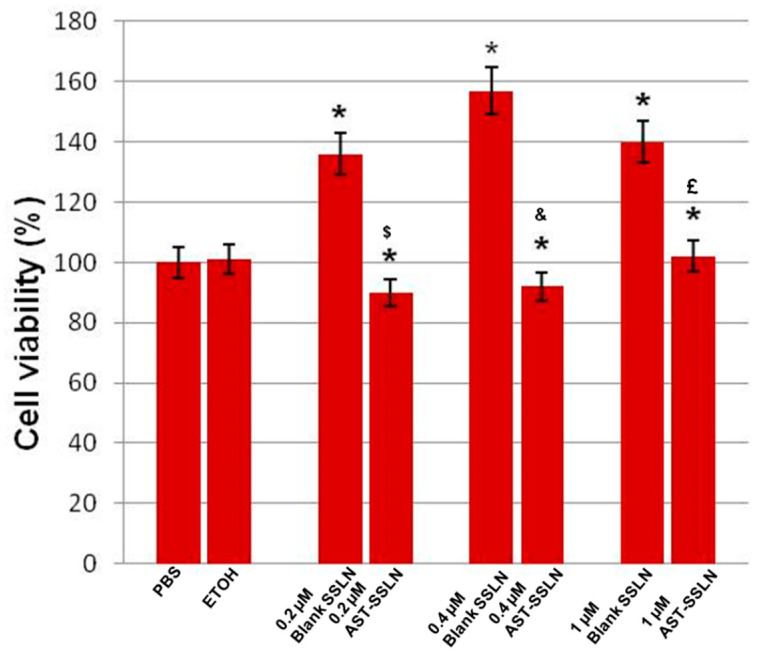
Percentage of cell viability in MCF-7 exposed to 0.005% of PBS (phosphate buffer saline solution), 0.005% ETOH (ethanol), Blank-SSLN, and AST-SSLN (0.2, 0.4, and 1 µM) for 24 h. All the values shown represent the average ± S.D. of three independent experiments performed in triplicate. * *p* < 0.05 vs. the respective controls; ^$^ *p* < 0.05 0.2 µM AST-SSLN vs. 0.2 µM Blank-SSLN; ^&^ *p* < 0.05 0.4 µM AST-SSLN vs. 0.4 µM Blank-SSLN; ^£^ *p* < 0.05 1 µM AST-SSLN vs. 1 µM Blank-SSLN.

Figure 8 shows the effect of the treatment of MCF-7 cell line cultures with blank-SSLN and AST-SSLN at different concentrations (0.2, 0.4, and 1 μM) for 24 h. No significant differences between PBS and ethanol-treated MCF-7 cell line cultures were found, hence they were used as controls. The percentage of inhibition of cell viability was compared with the controls taken as 100%. A significant increase in the percentage of cell viability in blank SSLN at all concentrations (0.2, 0.4, and 1 µM) was observed when compared with the controls. The effect was more evident at 0.4 µM for 24 h. This increase could be related to the lipid formulation exerting a protective effect on breast cancer cell membranes. This hypothesis is supported by other findings [54,55]. In contrast, when the cells were exposed for 24 h to the same concentration of AST-SSLN, a significant reduction in the percentage of cell viability was found compared with the respective blank-SSLN (Figure 8). The effect appeared more evident in MCF-7 cell line cultures treated with 0.2 µM AST-SSLN, when compared with the respective blank SSLN. Thus, the optimal concentration of AST-SSLN was 0.2 µM for 24 h. The effect of AST-SSLN in MCF-7 cell line might be due to the activation of the apoptotic pathway in cancer cells.

### 3.7. ORAC Assay 

**Figure 9:**
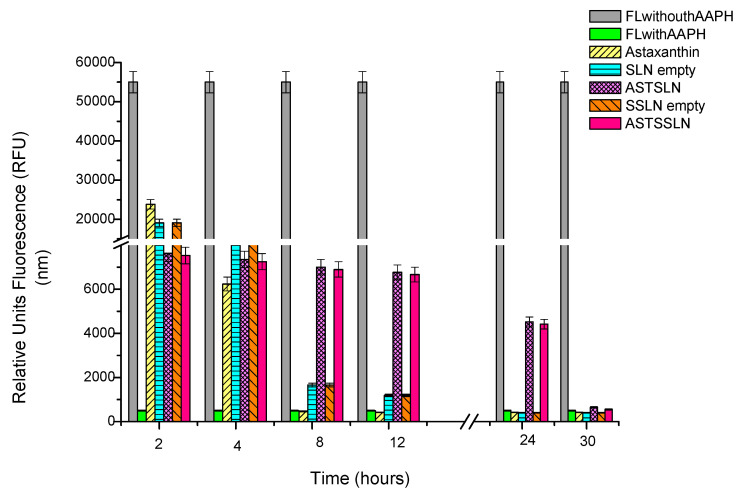
ORAC assay of free AST, empty SLN and SSLN, AST-SLN, and AST-SSLN. FL = fluorescein; FL + AAPH = fluorescein solution containing AAPH (2,2′-azobis(2-methylpropionamidine) dihydrochloride). Data represent the mean of three independent experiments ± SD.

The antioxidant capacity of AST, free and encapsulated into SLN and SSLN, was evaluated using the oxygen radical absorbance capacity (ORAC) assay, a widely used in vitro test used to measure the antioxidant capacity of natural compounds [56,57]. The AST ability to inhibit quenching of the fluorescence probe induced by free radicals was expressed in terms of preservation of the fluorescence signal over time. The obtained values show that at 4 h, all the formulations (blanks, AST-SLN and AST-SSLN) had a high fluorescence value compared to the untreated control (FL with AAPH) which implies that they are all protective, since they preserve fluorescein from the degradation by AAPH-derived peroxyl radicals. At 12 h, AST-SSLN and AST-SLN had a much higher fluorescence value (6770 and 6760 nm respectively) than free AST (620 nm). In accordance with the literature [58], these results suggest that encapsulation of AST into lipid nanoparticles (SSLN and SLN) preserve the antioxidant capacity of AST for a longer time (30 h) and probably maintains its stability (Figure 9). Since the obtained results on AST-SLN and AST-SSLN were similar, we focused the subsequent study on the stealth systems AST-SSLN.

### 3.8. UV Stability Assay

**Figure 10:**
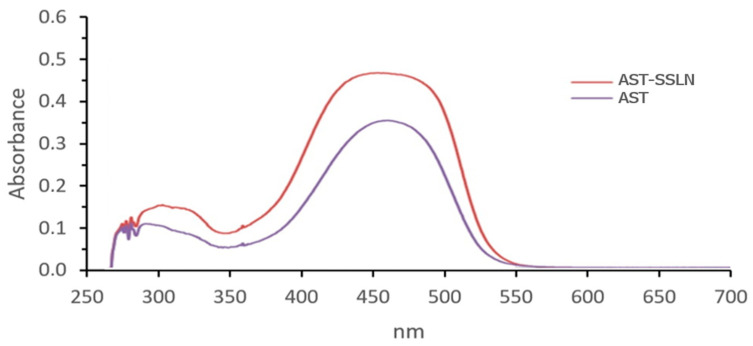
UV-absorption spectra of AST alone and once loaded in SSLN after exposure to 275 kJ/m^2^ UVA.

The photoprotective effect of the lipid nanoparticles against UVA radiation was evaluated using the UV stability assay as previously reported [59]. As shown in Figure 10, after UVA exposure AST loaded in SSLN retains the same spectral profile as free AST but with a higher absorbance. This indicates that the lipid shell of SSLN protects AST from photodegradation. This is attributable to the cis-trans photo-isomerization of AST [60].

**Figure 11:**
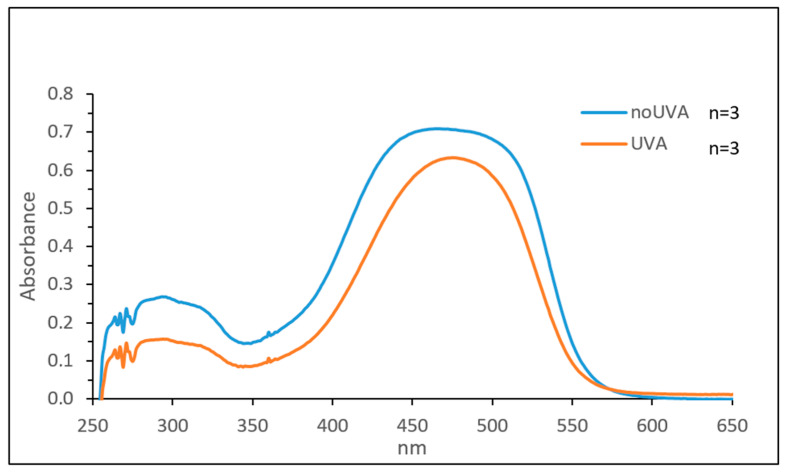
UV-absorption spectra of AST-SSLN before and after exposure to 275 kJ/m^2^ UVA.

Figure 11 shows the spectra of AST-SSLN before and after UVA exposure. There is a minimal decrease in absorbance, which confirms that lipid nanoparticle systems protect AST from potential UVA-induced photodegradation [60].

The promising results of the present work stimulate future research directions to test in vivo the efficacy of AST-SSLNs for the possible treatment of AD.

## 4. Conclusions

AST is a natural antioxidant that possesses therapeutic properties for the treatment of Alzheimer’s disease, although it shows poor bioavailability due to its high lipophilicity. In order to overcome these limits that compromise its therapeutic use, our attention focused on the development of innovative and efficient stealth carriers (SSLN) loaded with AST, capable of avoiding the defense line represented by the macrophages and of improving the drug stability, in order to achieve good bioavailability in the brain. The best strategy to realize SSLN, suitable for parenteral administration, was to coat the AST-SLN with surfactant polysorbate 80 (AST-SSLN).

AST-SSLN were prepared by solvent-diffusion technique showing a good mean particle size suitable for parenteral administration (<200 nm). In terms of stability, AST-SSLN showed an acceptable stability during six months of storage. The lipid formulation did not produce toxic effects on the cell lines studied and AST-SSLN showed a greater antioxidant capacity over time than free AST. The protective effect of SSLN was further demonstrated by the UV stability assay confirming that the lipid shell protected AST from photodegradation. Therefore, SSLNs could be regarded as a promising carrier for AST in the treatment of CNS disorders, through systemic administration.

## Figures and Tables

**Table 1:** Mean particle size (Z-Ave), polydispersity index (PDI), and zeta potential (ZP) of SLN and SSLN unloaded and loaded with AST.

Formulation	Z-Ave [nm ± SD]	PDI [–] ± SD	ZP [mV ± SD]
Blank SLN	136.0 ± 0.2	0.27 ± 0.1	−20.4 ± 0.2
AST-SLN	111.1 ± 0.2	0.33 ± 0.2	−16.2 ± 0.2
Blank SSLN	154.2 ± 0.3	0.29 ± 0.2	−18.2 ± 0.3
AST-SSLN	130.8 ± 0.2	0.32 ± 0.3	−13.0 ± 0.4

## Data Availability

Data is available on the request from the corresponding author.
