# Correction: Santonocito et al. Astaxanthin-Loaded Stealth Lipid Nanoparticles (AST-SSLN) as Potential Carriers for the Treatment of Alzheimer’s Disease: Formulation Development and Optimization. *Nanomaterials* 2021, *11*, 391

**DOI:** 10.3390/nano13243088

**Published:** 2023-12-06

**Authors:** Debora Santonocito, Giuseppina Raciti, Agata Campisi, Giovanni Sposito, Annamaria Panico, Edy Angela Siciliano, Maria Grazia Sarpietro, Elisabetta Damiani, Carmelo Puglia

**Affiliations:** 1 Department of Drug Science and Health, University of Catania, Viale Andrea Doria 6, 95125 Catania, Italy; debora.santonocito@outlook.it (D.S.); racitigi@unict.it (G.R.); agcampisi@gmail.com (A.C.); giovanni.sposito@hotmail.it (G.S.); panico@unict.it (A.P.); edysiciliano@hotmail.it (E.A.S.); mg.sarpietro@unict.it (M.G.S.); 2 Department of Life and Environmental Sciences, Polytechnic University of Marche, 60121 Ancona, Italy; e.damiani@univpm.it

## Error in Figure

In the original publication [1], there was a mistake in Figure 3b as published. An error was committed during the preparation of the manuscript “Astaxanthin-Loaded Stealth Lipid Nanoparticles (AST-SSLN) as Potential Carriers for the Treatment of Alzheimer’s Disease: Formulation Development and Optimization”, in which the image in Figure 3b is similar to Figure 2a from another article, “Curcumin Containing PEGylated Solid Lipid Nanoparticles for Systemic Administration: A Preliminary Study” published in *Molecules*. The corrected Figure 3b appears below. 


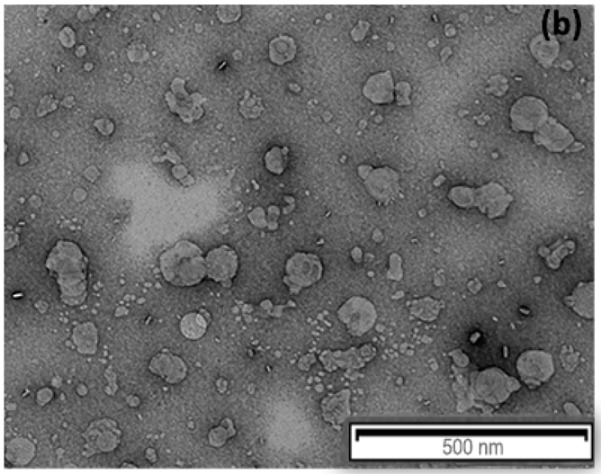


The authors apologize for any inconvenience caused and state that the scientific conclusions are unaffected. This correction was approved by the academic editor. The original publication has also been updated.
